# Manic episode following SARS-COV-2 Infection

**DOI:** 10.1192/j.eurpsy.2022.1316

**Published:** 2022-09-01

**Authors:** Z. Bencharfa, Y. Amara, N. El Moussaoui, S. Belbachir, A. Ouanass

**Affiliations:** University Hospital Center Ibn Sina, Ar-razi Psychiatric Hospital, Salé, Morocco

**Keywords:** manic episode, Covid-19

## Abstract

**Introduction:**

In December 2019, infection with the novel coronavirus (SARS-CoV-2) was first reported in the city of Wuhan, China. Although generally recognized for its often fatal respiratory problems, other neuropsychiatric complications are receiving increasing attention.

**Objectives:**

We will try through a clinical case to explain the psychiatric disorders in the context of this infection, and to highlight the two main explanatory theories of psychiatric disorders, in relation with the SARS-Cov-2 infection.

**Methods:**

We report here a case of SARS-CoV-2 infection in a 54-year-old female patient with no specific pathological history, including psychiatric, who presented a fever, anosmia, and asthenia in the absence of any respiratory signs. Her PCR came back positive and her chest CT scan was normal.The patient was treated with paracetamol with vitamin C,with good clinical improvement. She came 15 days later to the psychiatric emergency room with psychomotor excitement. The patient was motorically unstable, could not hold still, her mimicry was hypermobile, contact with her was familiar, she was logorrheic with flight of ideas,she verbalized multiple projects, her mood was euphoric and her sleep was disturbed. Her blood tests were unremarkable.

**Results:**

The diagnosis of manic episode was retained, and the patient was put on Olanzapine 10 mg, sodium Valproate 1g and Lorazepam 2.5 mg in degression with good clinical improvement.

**Conclusions:**

Although the data in the literature remain scarce concerning the impact of this virus on mental health, we will try through this clinical case to explain the psychiatric disorders in the context of this viral epidemic, due to stress and inflammation.

**Disclosure:**

No significant relationships.

